# A PNPase Dependent CRISPR System in *Listeria*

**DOI:** 10.1371/journal.pgen.1004065

**Published:** 2014-01-09

**Authors:** Nina Sesto, Marie Touchon, José Marques Andrade, Jiro Kondo, Eduardo P. C. Rocha, Cecilia Maria Arraiano, Cristel Archambaud, Éric Westhof, Pascale Romby, Pascale Cossart

**Affiliations:** 1 Unité des Interactions Bactéries-Cellules, Institut Pasteur, Paris, France; 2 INSERM, U604, Paris, France; 3 INRA, USC2020, Paris, France; 4 Unité de Génomique Evolutive des Microbes, Institut Pasteur, Paris, France; 5 CNRS, UMR3525, Paris, France; 6 Control of Gene Expression, Instituto de Tecnologia Química e Biológica, Oeiras, Portugal; 7 Architecture et Réactivité de l'ARN Université de Strasbourg, CNRS, Strasbourg, France; Institute of Molecular and Cell Biology (IMCB), A*STAR, Singapore

## Abstract

The human bacterial pathogen *Listeria monocytogenes* is emerging as a model organism to study RNA-mediated regulation in pathogenic bacteria. A class of non-coding RNAs called CRISPRs (clustered regularly interspaced short palindromic repeats) has been described to confer bacterial resistance against invading bacteriophages and conjugative plasmids. CRISPR function relies on the activity of CRISPR associated (*cas*) genes that encode a large family of proteins with nuclease or helicase activities and DNA and RNA binding domains. Here, we characterized a CRISPR element (RliB) that is expressed and processed in the *L. monocytogenes* strain EGD-e, which is completely devoid of *cas* genes. Structural probing revealed that RliB has an unexpected secondary structure comprising basepair interactions between the repeats and the adjacent spacers in place of canonical hairpins formed by the palindromic repeats. Moreover, in contrast to other CRISPR-Cas systems identified in *Listeria*, RliB-CRISPR is ubiquitously present among *Listeria* genomes at the same genomic locus and is never associated with the *cas* genes. We showed that RliB-CRISPR is a substrate for the endogenously encoded polynucleotide phosphorylase (PNPase) enzyme. The spacers of the different *Listeria* RliB-CRISPRs share many sequences with temperate and virulent phages. Furthermore, we show that a *cas*-less RliB-CRISPR lowers the acquisition frequency of a plasmid carrying the matching protospacer, provided that *trans* encoded *cas* genes of a second CRISPR-Cas system are present in the genome. Importantly, we show that PNPase is required for RliB-CRISPR mediated DNA interference. Altogether, our data reveal a yet undescribed CRISPR system whose both processing and activity depend on PNPase, highlighting a new and unexpected function for PNPase in “CRISPRology”.

## Author Summary

CRISPR-Cas systems confer to bacteria and archaea an adaptive immunity that protects them against invading bacteriophages and plasmids. In this study, we characterize a CRISPR (RliB-CRISPR) that is present in all *L. monocytogenes* strains at the same genomic locus but is never associated with a *cas* operon. It is an unusual CRISPR that, as we demonstrate, has a secondary structure consisting of basepair interactions between the repeat sequence and the adjacent spacer. We show that the RliB-CRISPR is processed by the endogenously encoded polynucleotide phosphorylase enzyme (PNPase). In addition, we show that the RliB-CRISPR system requires PNPase and presence of *trans* encoded *cas* genes of a second CRISPR-Cas system, to mediate DNA interference directed against a plasmid carrying a matching protospacer. Altogether, our data reveal a novel type of CRISPR system in bacteria that requires endogenously encoded PNPase enzyme for its processing and interference activity.

## Introduction

*Listeria monocytogenes* is a gram-positive foodborne pathogenic bacterium that has evolved two distinct lifestyles: a saprophytic one, primarily in decaying vegetation and a parasitic one in the tissues of mammals and birds, causing a disease known as listeriosis. Infection in humans starts by the ingestion of contaminated food products that deliver the bacteria in the intestinal lumen. In the course of the infection of susceptible individuals e.g. elderly and pregnant women, *Listeria* can cross three barriers of the organism: the intestinal, blood-brain and feto-placental barriers, causing meningitis, encephalitis and abortion. The main and best studied regulator that orchestrates the *Listeria* infectious process is PrfA (Positive regulatory factor A), a transcription factor that activates expression of the major known virulence genes [1]. In addition to protein determinants contributing to infection, *Listeria* possesses a virulence gene repertoire that expands to non-coding RNA (ncRNAs) molecules [2]–[4].

Bacterial ncRNAs are key regulatory molecules of metabolic, physiological and pathogenic processes and can be generally classified in four groups: a) the RNA regulatory elements located in the 5′ untranslated regions (5′UTRs) which regulate the expression of the corresponding mRNAs through the binding of various factors, like proteins (e.g. CsrA) and small metabolites (riboswitches) or by sensing environmental cues like temperature (thermosensors); b) the trans-acting small RNAs (sRNAs) regulating one or several target mRNAs located elsewhere on the chromosome; c) the sRNAs that sequester RNA-binding proteins; and d) the antisense transcripts (asRNAs), which overlap and are complementary to their target genes in the same genomic locus [5]. A novel class of non-coding RNAs, named CRISPRs (clustered regularly interspaced short palindromic repeats) has been shown to mediate bacterial adaptive immunity against invading bacteriophages and conjugative plasmids. A CRISPR is defined by the alternating array of identical 20–40 nucleotides (nt) long repeat sequences, interspaced by non-repetitive spacer sequences. In the proximity of the locus, are usually found gene clusters called CRISPR-associated (*cas*) genes. *Cas* genes form 23–45 different gene families (depending on the classification), encoding diverse proteins with nuclease, helicase, integrase, polymerase or nucleotide-binding activities, which are involved in the different steps of CRISPR generation, maintenance, processing and the interference mechanism. Analysis of the various sets of *cas* genes has revealed that CRISPR-Cas system generally cluster into three basic types (Type-I, Type-II and Type-III) which are further divided into at least ten subtypes (Types IA–F, Types IIA–C and Types IIIA–B) [6], [7]. The first clue about CRISPR function was brought about by the discovery that the different spacers were homologous to bacteriophage and plasmid sequences [8]–[10]. It was thus hypothesized that CRISPRs could play a role in immunity against invading genetic elements, which was later experimentally demonstrated in several elegant studies, e.g. in *Streptococcus thermophilus* [11], *Escherichia coli* [12] and *Staphylococcus epidermidis* [13]. The mechanism of action underlying the whole process is still not entirely understood, but can be roughly divided in three major stages: i) CRISPR adaptation that occurs when bacteria first encounter the foreign invader after transformation, conjugation or transduction. The CRISPR system recognizes the foreign element and incorporates parts of its DNA into what becomes a new spacer in the CRISPR locus; ii) CRISPR expression that generates a long poly-spacer precursor RNA, which is then cleaved by Cas proteins producing smaller, mature RNAs (crRNAs). Each crRNA generally contains part of the repeat and a single spacer that serves as a guide for the sequence specific recognition of the foreign invader; iii) CRISPR interference mediated by mature target-specific crRNAs that, with the help of *cas* gene products, inactivate the foreign, bacteriophage or plasmid nucleic acid [14], [15].

In *Listeria*, 14 plasmids [16] and 11 bacteriophages [17] have been sequenced so far. Bacteriophages infecting *Listeria* belong to the *Siphoviridae* and *Myoviridae* families in the *Caudovirales* order. They are either temperate integrating in the host genome by site-specific recombination or virulent actively replicating and forming virion particles that subsequently lyse the host cell. Comparative genomic analysis of *Listeria* bacteriophages revealed that their genomes are highly mosaic, characterized by interspecies homology as well as homology to bacteriophages infecting *Bacillus*, *Enterococcus*, *Clostridium* and *Staphylococcus* [17], [18]. Prophages are considered as the major source of diversity within the *Listeria* genus [18] and can constitute up to 7% of the *Listeria* coding genes [19], [20].

Recently, CRISPR-Cas systems have started to be analyzed in *Listeria* [18], [21]. We previously described in *L. monocytogenes* strain EGD-e, a small CRISPR RNA (RliB) exhibiting five identical repeats interspaced by non-related spacer sequences of similar size. Strikingly, no *cas* genes were found either in the proximity of RliB or elsewhere in the *L. monocytogenes* EGD-e genome [22]. Despite the absence of Cas proteins, RliB is expressed and significantly upregulated in bacteria isolated from the intestinal lumen of gnotobiotic mice, in bacteria grown in the human blood, or bacteria exposed to hypoxia. More importantly, we showed that RliB is involved in *L. monocytogenes* virulence [4].

Here, we characterized the *cas*-less RliB-CRISPR by first determining its secondary structure and analyzing its processing. Furthermore, we undertook a search for RliB protein ligands, to address the molecular machinery underlying RliB processing in the absence of Cas proteins. By using two different protein affinity purification approaches, we showed that RliB binds and is a substrate for polynucleotide phosphorylase (PNPase), a bi-functional enzyme harboring a 3′ to 5′ exoribonuclease and 3′ polymerase activities [23]. Furthermore, we performed a global analysis of CRISPR-Cas systems in all sequenced *Listeria* genomes, revealing a striking ubiquity of the RliB-CRISPRs in *L. monocytogenes* strains. Surprisingly, RliB-CRISPRs are never associated with *cas* gene clusters and we could demonstrate that even in *Listeria* strains harboring a complete set of *cas* genes, RliB-CRISPRs are processed by PNPase. Finally, we carried out a functional assay for RliB-CRISPR and demonstrated it requires presence of the *cas* genes of a second CRISPR system to lower the acquisition frequency of a plasmid carrying the matching protospacer. Moreover, we show that PNPase is required for this DNA interference activity. Together, our data highlight a novel type of CRISPR system that relies on the activity of PNPase, highlighting a new role for this enzyme in bacteria.

## Results

### RliB is a CRISPR RNA expressed and processed in the absence of Cas proteins

**Figure 1:**
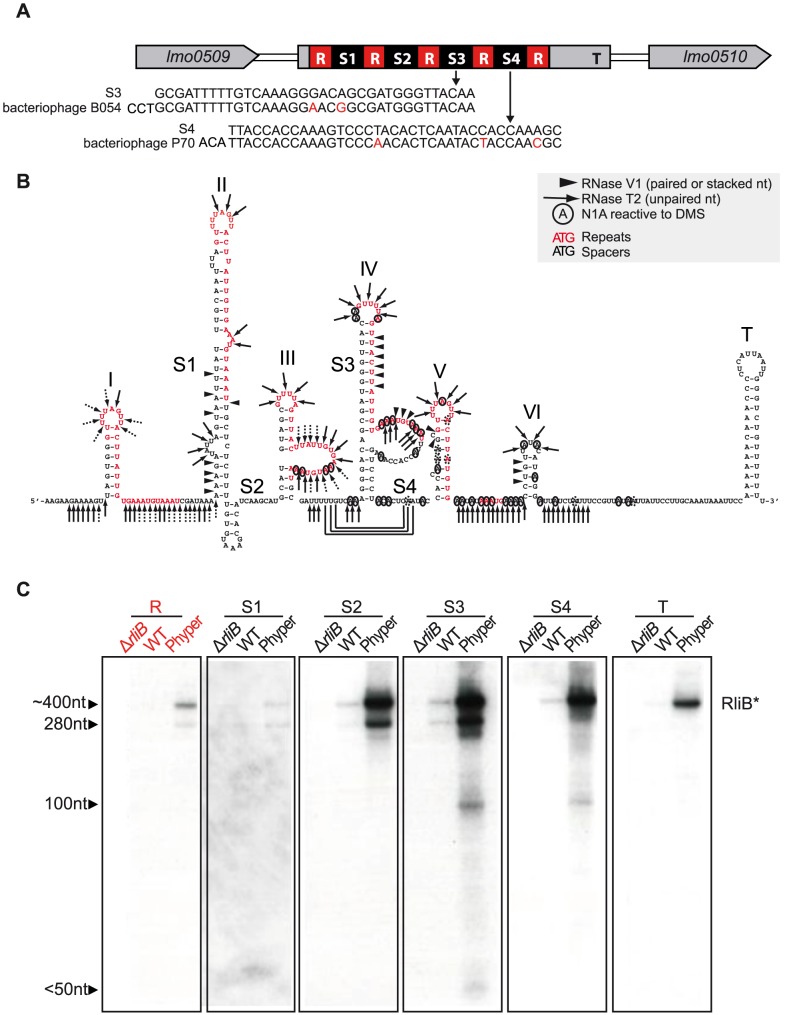
RliB in *L. monocytogenes* EGD-e. A) Schematic representation of the RliB locus. RliB is located between genes *lmo0509* and *lmo0510*. It is composed of 5 identical repeat sequences (R), 4 spacer sequences (S1 to S4) and the terminator (T). The sequence alignments between the spacers and bacteriophage genomes are shown. B) RliB secondary structure. Repeat sequences are highlighted in red, spacers are numbered S1 to S4, the hairpin structures are numbered I to VI and a terminator hairpin is indicated T. C) RliB processing. Northern blot on total RNAs extracted from bacteria deleted for RliB (Δ*rliB*), wild type bacteria (WT) and bacteria overexpressing RliB (Phyper RliB). Expression of RliB was detected using P^32^-5′ labelled probes complementary to different parts of the RliB molecule: repeat (R), spacer 1 (S1), spacer 2 (S2), spacer 3 (S3), spacer 4 (S4), 5′end and the terminator (T).

In *L. monocytogenes* EGD-e, RliB is located between the genes *lmo0509* and *lmo0510* that encode a protein similar to phosphoribosyl pyrophosphatase and a hypothetical protein, respectively. Its primary sequence resembles a typical CRISPR. It is composed of 5 identical 29 nt repeat sequences (GTTTTAGTTACTTATTGTGAAATGTAAAT) interspaced by four 35–37 nt long spacer sequences (S1, S2, S3 and S4 in the Figure 1A). The spacer 3 (S3) has identity with the *Listeria* temperate bacteriophage B054 sequence and spacer 4 (S4) identity to *Listeria* virulent bacteriophage P70 sequence [17], [24]. We analyzed the secondary structure of the full length RliB that is detectable *in vivo*, using RNase V1 (specific for helical regions), RNase T2 (specific for unpaired nucleotides with a preference for adenines) and dimethylsulfate (which methylates N1 of adenine and N3 of cytosine) (Figure S1). The secondary structure of RliB, that explains most of the probing data, involves six stem-loop structures among which five contain a GUUUU motif within the loops, followed by a hairpin terminator at the 3′ end (Figure 1B). In contrast to CRISPR systems where the repeat sequences form independent and stable palindromic structures [25], the RliB hairpin structures are mostly formed by base pairings between the repeat sequences and the adjacent spacer sequence. These data suggest that RliB structure largely depends on the nature of the incoming spacer DNA.

We had previously noticed that RliB in *L. monocytogenes* EGD-e is processed to a smaller fragment [22]. Here, we examined this processing by Northern blot analysis of total RNA isolated from the wild type (WT) *L. monocytogenes* EGD-e and bacteria expressing RliB from a constitutive promoter (Phyper-RliB). We used probes complementary to the repeat (R), to each unique spacer (S1, S2, S3 and S4) and to the 3′ end of the molecule including the terminator region (T) (Figure 1C). All the probes allowed detection of a 400 nt fragment, which corresponds to the full length RliB molecule. The probes for S1, S2, S3 and R regions detected an additional 280 nt long fragment. The probes for S3 and S4 regions showed a minor 100 nt long fragment and the probe for S3 region a fragment smaller than 50 nt.

Altogether, our data show that the RliB-CRISPR has a secondary structure largely determined by the interactions between each repeat and the adjacent spacer and, despite the absence of Cas proteins, it is processed and exists under two major forms: i) a 400 nt full length RliB molecule and ii) a shorter form of RliB molecule, approximately 280 nt long, comprising spacers S1, S2 and S3.

### RliB interacts with the Polynucleotide Phosphorylase (PNPase) enzyme

**Figure 2:**
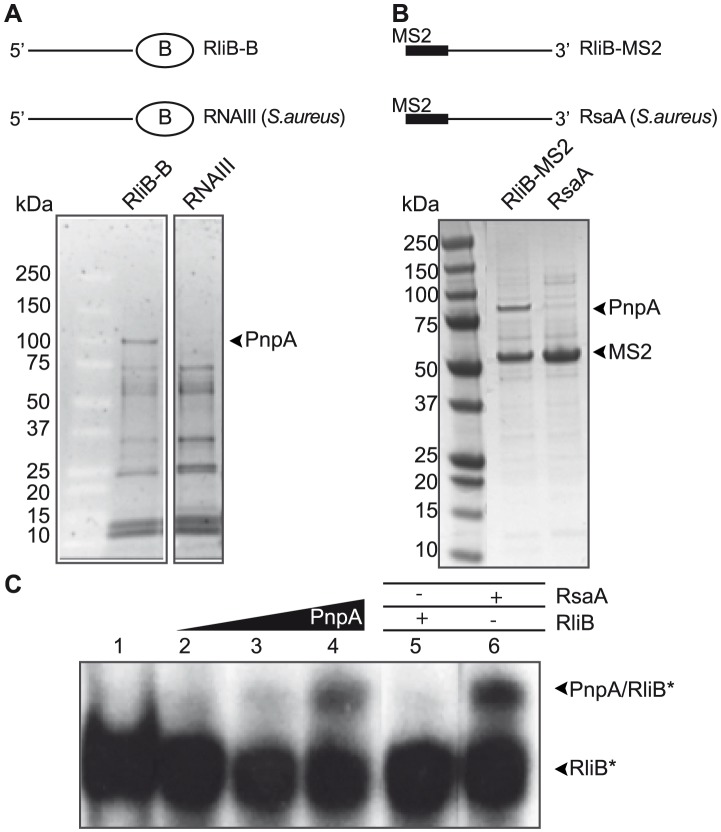
RliB interacts with PNPase. A) Protein affinity purification using 3′ biotinylated RliB. Two biotinylated bait RNAs were used: full length RliB (RliB-B) and a control RNA (*S. aureus* RNAIII). Coomassie stained SDS-PAGE gel of the two elution fractions is shown. B) Protein affinity purification using 5′ MS2 tagged RliB. Two MS2 tagged bait RNAs were used: full length RliB (RliB-MS2) and a control RNA (*S.aureus* RsaA). Coomassie stained SDS-PAGE gel of the fractions is shown. C) Interaction between purified PNPase and *in vitro* transcribed RliB. Gel retardation assay performed with P^32^-5′ labelled RliB (RliB) and increasing concentrations of the purified PNPase protein (lane 1, no PNPase added; lane 2, 100 nM PNPase; lane 3, 250 nM PNPase; lane 4, 400 nM PNPase). Competition experiments were performed in the presence of non-labelled RliB (lane 5, 400 nM RliB) and non-labelled RsaA (lane 6, 400 nM RsaA). In these experiments, the concentration of PNPase was 400 nM.

Considering the complete absence of *cas* genes in *L. monocytogenes* EGD-e strain, we hypothesized that the RliB-CRISPR processing is governed by another bacterial ribonuclease. To identify which enzymes are involved in this process, we first searched for proteins that interact with RliB using the affinity purification method with a tagged RliB molecule. Given that addition of a tag may perturb the folding of the bait-RNA molecule and/or change its accessibility, resulting in a loss of interaction with its binding partners, we used two strategies using two different tags added either at the 5′ or at the 3′ end of the bait-RNA. The first affinity purification was performed with the 3′-biotinylated full length RliB (RliB-B) and a control RNA, the quorum sensing induced RNAIII from *Staphylococcus aureus* (Figure 2A). In the second approach, we used as a bait RliB tagged at the 5′ end with two hairpin structures constituting the “MS2 binding sequence” (RliB-MS2), i.e. the RNA binding sequence of bacteriophage MS2 coat protein (MS2) (Figure 2B). Structure probing using enzymes show that the MS2-tag did not change the structure of RliB (Figure S1). The RliB-B or RliB-MS2 RNAs were bound to streptavidin or MBP-MS2 coated beads, respectively and incubated with total *Listeria* cell extracts. After extensive washing of unspecific proteins, the bound fraction was eluted and loaded on SDS-polyacrylamide denaturing gel. To verify the integrity of the bait-RNA, we also analyzed the eluted tagged RNA using polyacrylamide-urea gel electrophoresis. For both experiments, we detected a single and major protein band of approximately 78 kDa, specific to RliB-bound elution fractions (RliB-B and RliB-MS2) (Figures 2A,B). The protein was identified by mass spectrometry to be the *Listeria* polynucleotide phosphorylase (PNPase) encoded by gene *lmo1331* (*pnpA*), a bi-functional enzyme that acts as 3′-5′ exoribonuclease and a 3′-terminal polymerase [23], [26].

We then analyzed whether the interaction between RliB and PNPase is direct or requires another binding partner. The *L. monocytogenes* PNPase protein carrying 6 histidines at its C-terminal end was purified and binding experiments were carried out using gel retardation assays with *in vitro* transcribed P^32^-labeled full length RliB and increasing amount of the purified PNPase. Formation of a complex between RliB and PNPase was observed with 400 nM PNPase, showing that the interaction is direct and does not require another binding partner. To demonstrate the specificity of PNPase binding, competition experiments were done with various non-labelled RNAs. The addition of non-labelled RliB outcompeted the interaction between PNPase and P^32^-labeled RliB in contrast to the addition of a non-labelled control RNA from *S. aureus* (RsaA) that did not affect the complex formation (Figure 2C). Altogether, our results show that PNPase specifically interacts with RliB.

### RliB is a substrate for PNPase

**Figure 3:**
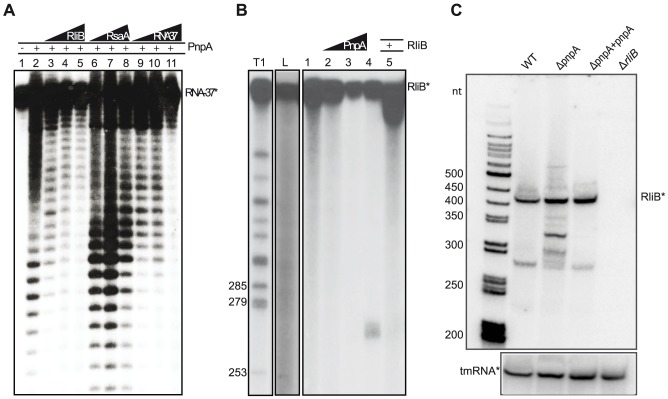
RliB is a substrate for PNPase. A) *In vitro* PNPase activity assay. A 37 nt short P^32^-labeled substrate RNA (RNA37*) was incubated alone (lane 1) or with 200 nM purified PNPase (lane 2). Three types of non-labeled competitor RNAs were then added: non-labeled RliB (lanes 3, 100 nM; lane 4, 200 nM; lane 5, 400 nM), non-labeled control RNA RsaA (lane 6, 100 nM; lane 7, 200 nM; lane 8, 400 nM) and non-labeled RNA37 substrate (lane 9, 100 nM; lane 10, 200 nM; lane 11, 400 nM). B) *In vitro* PNPase-mediated processing of RliB. On the left are indicated ladders T1 (RNase T1 hydrolysis) and L (alkaline hydrolysis). The full length P^32^-5′ labeled RliB (RliB*) was incubated with increasing concentrations of purified PNPase (lane 1, no PNPase; lane 2, 25 nM; lane 3, 100 nM; lane 4, 400 nM). Competition experiments were performed in the presence of 400 nM non-labelled RliB (lane 5). C) *In vivo* PNPase-mediated processing of RliB. Northern blot performed on the total RNAs extracted from the *L. monocytogenes* EGD-e wild type bacteria (WT), strain deleted for *pnpA* (Δ*pnpA*), complemented strain (Δ*pnpA-pnpA*) and strain deleted for RliB (Δ*rliB*). Expression of RliB was detected by the P^32^ labeled *in vitro* transcribed RNA probe complementary to the full length RliB. The membrane was reprobed with P^32^ labeled *in vitro* transcribed RNA probe complementary to the tmRNA.

PNPase is a bifunctional enzyme, which *in vivo* acts primarily as a 3′ to 5′ exoribonuclease of single stranded target RNAs [26]. We investigated if RliB is a substrate of PNPase. We first verified the activity of the purified PNPase protein and performed an *in vitro* assay where a 37 nt P^32^-end labeled substrate RNA (RNA37) was incubated alone or with 200 nM purified PNPase (Figure 3A). The presence of PNPase resulted in the degradation of the 5′ end P^32^-labeled RNA37 while no cleavage reaction was observed using a P^32^-pCp 3′ end labeled RNA37 (results not shown). These data demonstrate that the purified protein is active and able to degrade single-stranded RNA substrates. Three non-labeled competitor RNAs were then added to the reaction; i) a non-labeled RliB; ii) a non-labeled control RNA (RsaA); iii) and the non-labeled RNA37 substrate. As expected, the addition of non-labeled RNA37 substrate decreased the cleavage reaction. Strikingly, the addition of 100 nM non-labeled RliB resulted in the loss of PNPase mediated RNA37 degradation, whereas addition of RsaA did not alter the degradation of RNA37, indicating that RliB acts as a competitive inhibitor of PNPase.

To investigate further the activity of PNPase on RliB, we incubated the full length 5′ end-labeled RliB with increasing concentrations of purified PNPase (Figure 3B). We observed on the gel the appearance of a band migrating around 270 nt, an RliB processing product generated by the PNPase-mediated degradation up to the stem-loop IV. This cleavage reaction was inhibited by the addition of the full length non-labeled RliB. Altogether, these data suggest that *in vitro*, PNPase is processing RliB until its exoribonuclease activity is stalled in the S4 repeat region.

To study the effect of PNPase on RliB *in vivo*, we constructed a *pnpA* deletion mutant (Δ*pnpA*) and compared by Northern blot the size of the RliB transcript in the Δ*pnpA* mutant and WT bacteria. In the absence of PNPase, two major bands migrating as 300 and 330 nt long RNAs, were observed. Upon complementation (Δ*pnpA-pnpA*), the RliB processing was restored, identical to that observed in the WT strain (Figure 3C). Our results thus strongly suggest that RliB is a substrate for PNPase *in vivo*.

### RliB-CRISPR is ubiquitous among *Listeria* strains

**Figure 4:**
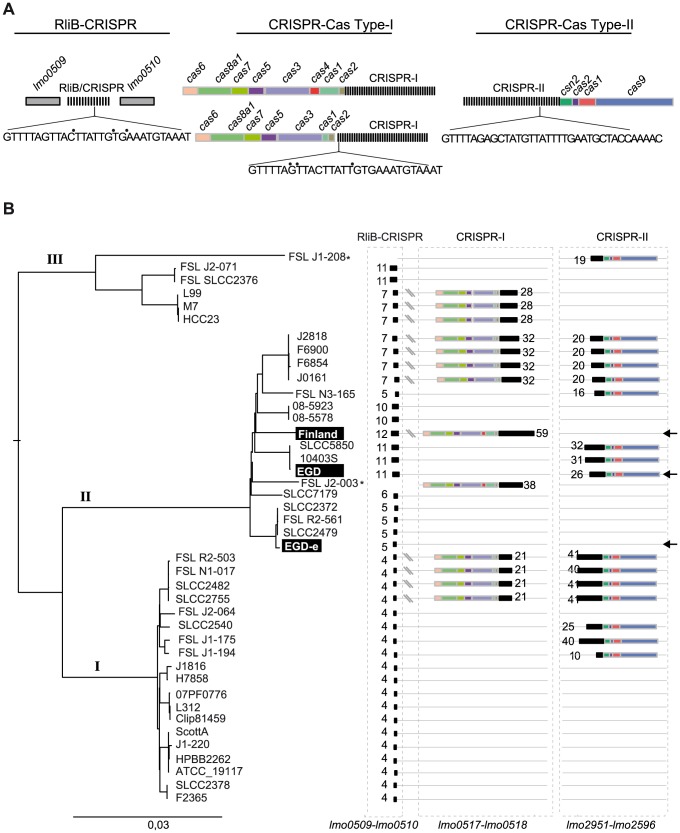
CRISPR-Cas systems in *L.monocytogenes* genomes. A) The three types of CRISPR-Cas systems found in *L.monocytogenes*. The sequences of the repeats are given. Points (•) indicate sites of repeat variability among *Listeria* strains. B) CRISPR-Cas systems in complete and draft *L. monocytogenes* genomes of the lineages I, II and III. For two strains indicated by an *, the sequencing results are too preliminary with a high number of contigs and RliB was not detected. However, these strains were included in the spacer analysis.

CRISPR arrays are thought to evolve rapidly in prokaryotic genomes [14], [27], [28]. Therefore, we investigated the presence of RliB in other *Listeria* strains. For this, we searched for CRISPRs in 29 complete and 17 draft *Listeria* genomes (Table S1). As mentioned in the introduction, the highly diverse CRISPR-Cas systems are classified into three main types (I, II and III) each including several subtypes [6]. In *Listeria*, we found two types of CRISPR-Cas systems: i) CRISPR-Cas systems type-I (subtype I-B) with the *cas* operon composed of *cas6-cas8a1-cas7-cas5-cas3-cas1*, including also in some cases *cas4*, associated with the repeat sequence GTTTTAGTTACTTATTGTGAAATGTAAAT that is almost identical to the repeat of RliB-CRISPR; ii) CRISPR-Cas systems type-II (subtype II-A) associated with *csn2-cas2-cas1-cas9* operon and the repeat sequence GTTTTGTTAGCATTCAAAATAACATAGCTCTAAAAC (Figure 4A).

CRISPR-I is present at the locus between *lmo0517* and *lmo0510* in 7 complete *L. monocytogenes* genomes, 10 draft *L. monocytogenes* genomes, in *Listeria seeligeri* and *Listeria ivanovii* (Figure 4B and Table S1) and it is always associated with a type-I *cas* operon located in close proximity. The CRISPR-II was detected between *lmo2591* and *lmo2596* in 6 complete *L. monocytogenes* genomes, 9 draft genomes and in *Listeria innocua* (Figure 4B, Table S1). The CRISPR-II is also found exclusively associated with type-II *cas* operon. The tight association of CRISPR-I and CRISPR-II with type-I and type-II *cas* genes, suggests that the function of those CRISPRs is dependent on the activity of the corresponding Cas protein machinery.

In contrast to CRISPR-I and CRISPR-II that are found in about 30% of the complete *Listeria* genomes, the RliB-CRISPR is present at the same genomic locus in all analyzed complete and draft *L. monocytogenes* genomes as well as in other *Listeria* species (Figure 4B, Table S1). This suggests a stronger selective pressure on this element relative to the *cas*-associated CRISPRs. *In silico* structure prediction performed on three representative RliB-CRISPRs carrying different number of repeats revealed a putative secondary structure that is highly similar to that experimentally determined in *L. monocytogenes* EGD-e strain (Figure S2). *Cas* operons have not been detected in the close proximity to the RliB-CRISPRs. Furthermore, 14 complete *Listeria* genomes completely lack *cas* genes. The number of repeats in RliB-CRISPRs range from 1 to 11 and does not correlate with the presence or absence of *cas* genes elsewhere in the genome (Table S3). Together, the conservation of RliB-CRISPRs among *Listeria* strains suggests that they may have a function despite the absence of *Listeria* Cas proteins.

Although RliB-CRISPR and CRISPR-I have different pattern of conservation the two systems share almost identical repeat sequences (Figure 4A). To investigate the correlation between the two systems, we compared their putative leader and upstream sequences. Multiple alignments of the DNA fragment preceding the identified RliB-CRISPR and CRISPR-I systems revealed a striking homology (Figure S3A). More interestingly, the putative leader sequences harbour a highly conserved sequence homologous to RpoD dependent promoter, that was previously reported upstream of RliB in the *L. monocytogenes* EGD-e strain [22] (Figure S3B). High homology of the repeats and the leader sequences of RliB-CRISPR and CRISPR-I systems suggest a possible close relationship between the two systems.

### RliB-CRISPRs in *Listeria* strains carrying *cas* genes are also substrates for PNPase

**Figure 5:**
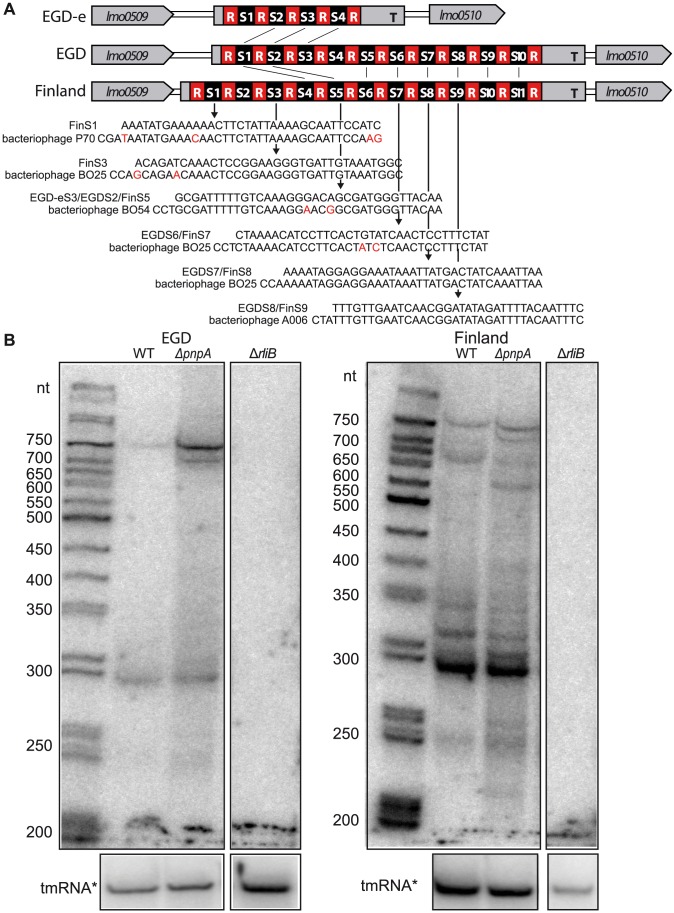
RliB-CRISPRs in EGD and Finland *L. monocytogenes* strains. A) Schematic representation of the loci harbouring RliB-CRISPR in *L.monocytogenes* EGD-e, EGD and Finland strains. In all three strains, RliB-CRISPR is located between genes *lmo0509* and *lmo0510*. Spacers that are identical between the strains are connected with black lines (S1EGD = S4Fin, S2EGD = S5Fin, S5EGD = S6Fin, S6EGD = S7Fin, S7EGD = S8Fin, S8EGD = S9Fin, S9EGD = S10Fin and S10EGD = S11Fin) and the sequence alignments between the spacers and bacteriophage genomes are shown below. B) Processing of the RliB-CRIPSRs. On the left is shown a northern blot performed on the total RNAs extracted from the wild type *L. monocytogenes* EGD strain (WT) and its isogenic mutants deleted for *pnpA* (Δ*pnpA*-EGD) or RliB-CRISPR (Δ*RliB*-EGD). On the right is shown a northern blot performed on the total RNAs extracted from the wild type *L. moncytogenes* Finland strain (WT) and its isogenic mutants deleted for *pnpA* (Δ*pnpA*-Fin) or RliB-CRISPR (Δ*RliB*-Fin). Expression of RliB-CRISPR was detected with the P^32^ labeled *in vitro* transcribed RNA probe complementary to the spacers S5 to S10 in EGD, and S6 to S11 in the Finland strain. The membranes were reprobed with P^32^ labeled *in vitro* transcribed RNA probe complementary to the tmRNA.

To investigate if the PNPase-mediated processing of RliB-CRISPR is specific to *L. monocytogenes* EGD-e strain or is more general, we examined if PNPase is also involved in RliB-CRISPR processing in *Listeria* strains containing a complete set of *cas* genes. We constructed a *pnpA* deletion mutant in the *L. monocytogenes* Finland strain (Δ*pnpA*-Fin), which carries a complete CRISPR-Cas system type I and in the *L. monocytogenes* EGD strain (Δ*pnpA*-EGD), which has a complete CRISPR-Cas system type II. We also constructed the deletion mutants for the RliB-CRISPR in the same strains (Δ*rliB-CRISPR*-Fin and Δ*rliB-CRISPR*-EGD, respectively). The RliB-CRISPR processing was examined by northern blot in the corresponding strains (Figure 5).

**Figure 6:**
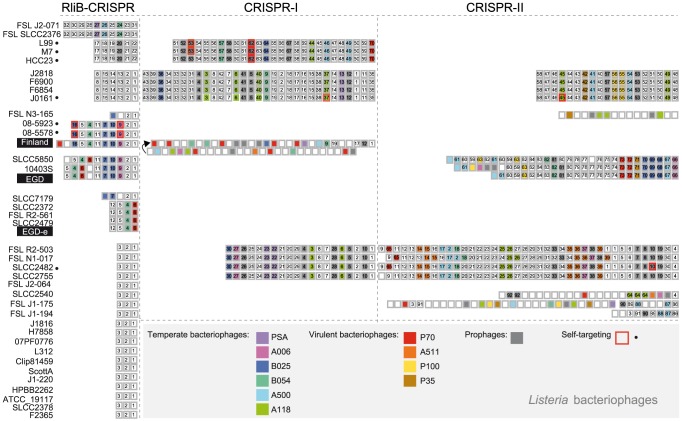
Spacer composition of CRISPR arrays in *L. monocytogenes*. CRISPR systems present in complete and draft *L. monocytogenes* genomes are represented. For each *L. monocytogenes* strain spacers belonging to RliB-CRISPR, CRISPR-I and CRISPR-II are shown. Boxed numbers represent individual spacers conserved within an CRISPR array among the represented strains (for the RliB-CRISPR: 1-32, for the CRISPR-I: 1-70, for the CRISPR-II: 1- 92). Unique spacers are represented as not numbered boxes. Spacers matching bacteriophage sequences available in the Genbank are coloured according to the type of bacteriophage they target. The color code and corresponding bacteriophages are represented in the bottom part of the figure. Spacers matching prophages are highlighted as gray boxes. Self-targeting spacers, identified in complete *Listeria* genomes are highlighted with red box and the strain carrying the spacer is highlighted (•).

The RliB-CRISPR in EGD strain (RliB-CRISPR-EGD) is composed of 11 identical repeats and 10 spacer sequences among which spacers S2, S6, S7 and S8 share similarity to *Listeria* temperate bacteriophages B054, B025 and A006 (Figure 5A, 6). In the WT EGD strain, RliB-CRISPR is expressed as a 750 nt long RNA that is processed into shorter fragments with the major processed form being 280 nt long. In the absence of PNPase, the total amount of full-length RliB-CRISPR-EGD increased and the transcript processing changed compared to the WT bacteria, i.e. we observed additional bands with a major one of 700 nt (Figure 5B).

The RliB-CRISPR in the Finland strain (RliB-CRISPR-Fin) is composed of 12 identical repeats and 11 spacer sequences among which 8 spacers are shared with RliB-CRISPR-EGD. Spacers S1, S3, S5, S7, S8 and S9 show high similarity to sequences in *Listeria* bacteriophages P70, B025, B054 and A006 (Figure 5A, 6). The full-length RliB-CRISPR-Fin is expressed in the WT bacteria, as a 780 nt long RNA that it is processed to several shorter fragments with the 280 nt being again the most abundant form. In the absence of PNPase, RliB-CRISPR-Fin processing changed as additional bands are observed compared to the WT bacteria (Figure 5B).

Together, our results suggest that PNPase contributes to the RliB-CRISPR processing *in vivo*, independently of the presence of either CRISPR-Cas system type I in the *L. monocytogenes* Finland strain, or the presence of CRISPR-Cas system type II in the *L. monocytogenes* EGD strain.

### Spacers of the RliB- CRISPRs are directed against *Listeria* bacteriophages

We analyzed CRISPR spacers to compare the putative functions of *cas*-less RliB-CRISPR and *cas*-associated CRISPR-I and CRISPR-II. In total, we identified 978 spacers that correspond to 348 unique sequences (Table S2). These were used to search for the similarity with the sequences of all complete prokaryote, plasmid and virus genomes available in the Genbank as well as the sequences of integrated temperate bacteriophages (prophages) identified in complete *Listeria* genomes (Figure 6, Table S3).

We identified 142 (41%) spacers that share identity to bacteriophages known to infect *Listeria* species (Figure 6). They match sequences detected in 6 temperate (B054, B052, A118, A500, A006, PSA), 4 virulent phages (A115, P35, P70, P100) as well as 35 distinct prophages found in complete *Listeria* genomes (Figure S4). Overall, we found matching protospacers for 33% RliB-CRISPR spacers, 41% CRISPR-I spacers and 42% CRISPR-II spacers (Figure 6, Table S3). RliB-CRISPR and CRISPR-I systems share an identical protospacer adjacent motif (PAM) CCA at the 5′ of the protospacer, in contrast to CRISPR-II harboring NGG at the 3′ of the protospacer (Figure S5). Numerous spacers showed 100% identity with viral sequences (14% RliB-CRISPR spacers, 15% CRISPR-I spacers and 24% CRISPR-II spacers). None of the spacers matched bacterial (excluding prophages) or plasmid sequences. The high abundance of spacers perfectly matching bacteriophages in the RliB-CRISPRs and in the CRISPR-I and CRISPR-II, suggests that both *cas*-less and *cas*-associated CRISPR-Cas systems have a role in the immunity against bacteriophages.

To investigate the nature of the phage nucleic acid potentially targeted by the RliB-CRISPR, CRISPR-I and CRISPR-II systems, we first examined the orientation of the protospacers in respect to the corresponding spacers and then the function of the phage genes where the protospacers are located. Protospacers targeted by CRISPR-I and CRISPR-II systems originate both from sense and antisense DNA strand and are equally distributed along the phage genome, which suggests that these systems target phage DNA (Table S4, S5, Figure S6). Among 13 protospacers targeted by RliB-CRISPR, 9 protospacers are in the antisense orientation, 3 are positioned in intergenic regions and 2 are in the sense orientation. Moreover, among 11 protospacers for which the function of the targeted gene is known, 10 protospacers are located in the late phage genes encoding DNA packaging and structural proteins (Table S6, Figure S6). The occurrence of both sense and antisense oriented protospacers suggests RliB-CRISPR most probably targets DNA. However, higher occurrence of the antisense oriented protospacers and more interestingly, specificity for the function of the targeted bacteriophage gene suggests that RliB-CRISPR could potentially have a function in RNA interference.

Furthermore, we identified genomes containing a number of spacers matching their own prophages. For example, *L. monocytogenes* strain EGD has prophage B025 (our unpublished data) and carries one RliB-CRISPR spacer (S7) and three CRISPR-II spacers (S21, S22, S23) that match the same prophage with up to 97% identity (Figure S4A,B). We also identified two RliB-CRISPR spacers, three CRISPR-I spacers and two CRISPR-II spacers in 9 *Listeria* genomes that correspond to prophages in the same genome with 100% identity. In two cases, the strains (*L. monocytogenes 08-5578* and *08-5923*) lack the *cas* genes and in one case the repeat flanking the self-targeting spacer carries a point mutation (*L. monocytogenes J0161*), suggesting that in three instances the spacers are presumably inactive (Table S7, Figure 6). The remaining spacers are either in *cas*-associated CRISPRs or in the RliB-CRISPR. Furthermore, the PAMS corresponding to self-targeted protospacers do not significantly deviate from the consensus (Table S7). These results show that spacers matching the protospacer located in the same bacterial chromosome do not necessarily have strong negative fitness effects.

### RliB-CRISPR DNA interference activity relies both on PNPase and *trans* encoded Cas proteins

**Figure 7:**
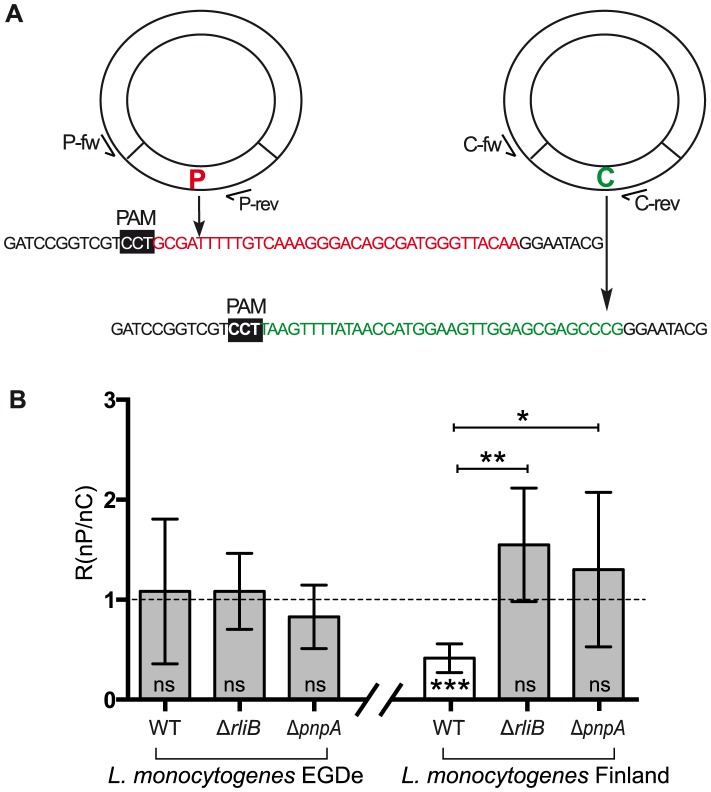
CRISPR DNA interference activity assay. A) Design of the plasmids. Two types of plasmids were used: i) protospacer plasmid (P) carrying the protospacer (highlighted in red) matching the spacer 3 in *L. monocytogenes* EGD-e strain RliB-CRISPR and spacer 5 in *L. monocytogenes* Finland strain RliB-CRISPR; ii) control plasmid (C) carrying the DNA fragment corresponding to the shuffled protospacer (highlighted in green). Black box marks the position of the protospacer adjacent motif (PAM). Position of the specific oligonucleotides used for the Q-PCR screening is indicated (P-fw, P-rev, C-fw, C-rev) B) Ratio (R). The number of colonies carrying plasmid P and the number of colonies carrying the plasmid C in the different *Listeria* strains and genetic backgrounds is represented as the ratio (R = n (colony P)/n (colony C). Shown are the mean and the standard deviation of five experiments performed for each bacterial strain. Two statistical tests were used: i) a t-test measuring if the ratio in the given strain is significantly different from 1 (*** - p = 0,0008; ns- not significant); ii) a t-test comparing the ratios of the WT strain and the Δ*rliB* and Δ*pnpA* strains (**- p = 0,0025; *- p = 0,0334).

To test if the *cas*-less RliB-CRISPR might provide *Listeria* with DNA interference activity, we designed an experiment using a conjugation system and two plasmids that differ in the presence or absence of protospacer: i) a protospacer plasmid (P) and ii) the control plasmid (C), as previously done by Almendros et al. [29] (Figure 7). The plasmid P carries a protospacer matching spacer 3 (S3) of the RliB-CRISPR in the *L. monocytogenes* EGD-e strain and spacer 5 (S5) of the RliB-CRISPR in the Finland strain. The plasmid C is identical to the plasmid P, but the protospacer sequence is shuffled *in silico* (C) and does not correspond to any known sequence in the NCBI database.

*Listeria* is not naturally competent and the plasmid transformation efficiency in this bacterium is very low in comparison to other bacteria such as *Bacillus* or *Streptococcus*. Moreover, the Δ*pnp* genetic background has a severe effect on bacterial growth, and plasmid transformation is even more difficult than in the WT strain. Therefore, the plasmids P and C were conjugated simultaneously *via Escherichia coli* S17 strains to *L. monocytogenes* EGD-e and Finland WT strains and their isogenic mutants deleted for RliB (Δ*rliB*) and PNPase (Δ*pnpA*). Quantitative PCR (Q-PCR) was used to determine the identity of the plasmids distributed among the transformants (see materials and methods). We then calculated for each individual strain the ratio (R) of the number of colonies carrying plasmid P and the number of colonies carrying plasmid C (R = nP/nC) (Figure 7B). The proportion of the transformants carrying the plasmid P for each experiment is an indication of the interference activity driven by the spacer, a lower proportion of transformants with the plasmid P (R<1) suggesting interference activity.

In the *L. monocytogenes* EGD-e in which no *cas* genes was identified, there is no significant difference in the R values between the strains carrying the RliB-CRISPR (WT) and the strains lacking either the RliB-CRISPR (Δ*rliB*-EGDe) or PNPase (Δ*pnpA*-EGDe), demonstrating that both plasmids are equally acquired and that the system is not able to provide any detectable DNA interference in the tested experimental conditions (Figure 7B). In contrast, in the *L. monocytogenes* Finland that bears an additional CRISPR-Cas Type-I system, the R ratio is significantly smaller than 1 in the WT strain whereas it reaches 1 in the strains lacking either the RliB-CRISPR (Δ*RliB*-Fin) or the PNPase (Δ*pnpA*-Fin). Thus, RliB-CRISPR can lower the plasmid P acquisition in the strain carrying the CRISPR-I system, suggesting that the RliB-CRISPR is able to use the *trans* encoded Cas proteins encoded by the CRISPR-I and confer to *Listeria* a DNA interference activity. Interestingly, PNPase is required for this process.

## Discussion

Since the initial discovery that CRISPR-Cas systems function as an adaptable prokaryotic immune system, the CRISPR research has been flourishing and biochemical insights into the CRISPR-Cas systems have increased dramatically over the past few years. However, these systems are extremely diverse and the function and molecular mechanism of many of them are still unknown. In particular, little is known about CRISPR-Cas type I-B, I-C, and I-D systems [30]. Here, we studied RliB-CRISPR that has an unusual secondary structure comprised of basepair interactions between the repeat sequence and the adjacent spacer. We showed that RliB-CRISPR is expressed and processed even in the complete absence of Cas proteins, and demonstrated this event occurs under the guidance of PNPase. The RliB-CRISPR spacers match numerous temperate and virulent *Listeria* bacteriophages and in the presence of CRISPR-Cas system Type-I, RliB-CRISPR lowers the frequency of a plasmid carrying a matching protospacer. In addition, we show that this DNA interference is dependent on the presence of PNPase. Overall, our data demonstrate that PNPase is involved in *Listeria* RliB-CRISPR processing and its DNA interference activity.

### Secondary structure of RliB

*In silico* analysis of the secondary structures of CRISPR repeats across bacterial and archaeal CRISPR-Cas systems suggested that some CRISPR repeats can form stable stem-loops due to the palindromic nature of their repeats, but that other lack any detectable conserved structure [25]. RliB repeats are only weakly palindromic and unlikely form a stable stem-loop structure. Here, we experimentally determined the secondary structure of RliB and surprisingly, discovered that RliB contained 6 hairpin structures formed mostly by base-pair interactions between the spacer sequences and the adjacent repeats, and with GUUU-rich apical loops (Figure 1B). The structure of RliB is thus dependent on the nature of the acquired spacer. *In silico* analysis of other representative RliB-CRISPRs showed their putative structures rely on the same principle, suggesting that base-pair interactions between the repeat and the spacer could be a common structural motif among RliB-CRISPRs (Figure S2). It is tempting to hypothesize that successful acquisition of a new spacer requires some degree of complementarity with the repeat. Spacer acquisition is the least understood step of CRISPR-Cas system function [31] and our data potentially highlight new aspects of the integration mechanism *via* homology with the repeat sequence.

### RliB-CRISPR processing by PNPase

It is generally accepted that CRISPR arrays require Cas proteins for their processing and activity. A first example of CRISPR-Cas system that does not rely solely on the Cas proteins but requires also the activity of endogenously encoded enzymes has been recently reported in *Streptococcus pyogenes*. In this case, a CRISPR array type-II is processed by the widely conserved endoribonuclease III and a small *trans*-acting RNA tracrRNA [32]. In addition, a recent study of Zhang et al [33] revealed a CRISPR in *Neisseria meningitidis*, where crRNAs are transcribed from promoters that are present within each repeat and require RNase III and *trans*-encoded tracrRNA-mediated processing for their maturation. Surprisingly, the maturation processing is dispensable for the CRISPR interference [33].

Here, we characterized a CRISPR array that is processed in a bacterium completely devoid of *cas* genes. In contrast to other CRISPRs, RliB-CRISPR is present in all sequenced strains of *L. monocytogenes*, even in other members of the genus and never co-localizes with *cas* operons. We demonstrated that RliB binds to and is a substrate for endogenously encoded PNPase, both in *cas*-less *Listeria* strains and in those encoding a complete set of *cas* genes elsewhere in the genome. Generally, PNPase degrades single-stranded RNA in a processive manner along the substrate until it stops, stalled by a stable RNA structure [23]. For instance, a hairpin structure in a bacteriophage mRNA can block the processivity of PNPase to protect the RNA against degradation [34]. It remains to be understood at the molecular level how PNPase specifically recognizes the RliB-CRISPR and how the progression of the enzyme stops.

In the three analyzed *L. monocytogenes* strains, RliB-CRISPRs is expressed as a full length molecule that is processed to several fragments out of which a 280 nt fragment is the most abundant form. The consistency of processing that is independent of the number of the repeat/spacer units suggests that the molecular mechanisms guiding the processing in the tested CRISPRs is conserved. Interestingly, in the bacteria deleted for *pnpA* (Δ*pnp*-EGDe, Δ*pnp* -EGD, Δ*pnp* -Fin), some processing still occurs, indicating there are other endogenously encoded ribonucleases contributing to this mechanism, particularly in the EGD-e strain that is devoid of *cas* genes (Figures 3C and 5B). *Listeria* encodes at least 17 different putative RNases identified by homology with closely related *Bacillus subtilis* [35]. Future work will have to determine which enzymes might function together with PNPase and also contribute to the CRISPR processing.

### The DNA interference activity of the RliB-CRISPRs

We showed that *cas*-less RliB-CRISPRs are rich in spacers matching virulent and temperate bacteriophages. In addition, a large fraction of those spacers have 100% matches with phages, strongly suggesting a function for RliB-CRISPR even in the absence of *cas* (Figure 6, Table S3). Accordingly, we showed that *cas*-less RliB-CRISPR lowers the acquisition of a plasmid carrying the corresponding protospacer, provided a CRISPR-I system is present (Figure 7). The RliB-CRISPR and CRISPR-I share many similar features; almost identical repeat sequence (Figure 4), homologous putative leader sequences (Figure S3) and identical PAM motifs (Figure S5), indicating that these two systems are closely related and are possibly functionally linked. It is thus not surprising that RliB-CRISPR can share the Cas machinery with the CRISPR-I to acquire the DNA interference activity, however future analysis will be required to establish the exact mechanism by which this crosstalk occurs.

More interestingly, the DNA interference activity of the RliB-CRISPR is also dependent on the presence of PNPase (Figure 7), indicating that the processing by this enzyme is important for the activity of the RliB-CRISPR. PNPase is a highly complex enzyme with 3′ to 5′ exoribonuclase and RNA polymerase activities being the most studied up to date. However, it was recently shown that PNPase can degrade single stranded DNA (ssDNA) and also catalyze template independent polymerization of dNDPs into 3′ends of ssDNA, which established a molecular model for the role of PNPase in DNA repair [36], [37]. In *Escherichia coli*, PNPase affects the stability of several regulatory sRNAs [38], [39]. Here, we hypothesize that *Listeria* PNPase, potentially with other endogenously encoded enzymes, may contribute to the RliB-CRISPR maturation. Alternatively, PNPase may affect the RliB-CRISPR RNA stability and turnover, and hence, regulate the levels of its mature form. Finally, PNPase dependent processing of the RliB-CRISPR and the DNA interference might be uncoupled activities. Hence, this complex enzyme could use different enzymatic activities to contribute to different processes. It will be also important to determine if PNPase is involved in other CRISPR-Cas system activities, such as new spacer acquisition. Currently, our data do not provide evidence on which form of RliB molecule is active in the DNA interference. These and other mechanistic details, such as the role of PAMs are to be determined in the future.

Noticeably, the RliB-CRISPR mediated DNA interference is not 100% effective. This might be the consequence of our experimental design or this CRISPR-Cas system did not evolve to eradicate the bacteriophage from a population but rather to fine-tune its copy number in the bacterial cytoplasm.

### The potential function of RliB-CRISPRs in the absence of Cas

Our functional assay showed that the RliB-CRISPR in the *L.monocytogenes* EGD-e strain that completely lacks *cas* genes, although processed by PNPase, is not able to provide DNA interference activity against a plasmid carrying a matching protospacer. This lack of activity is probably due to the absence of *trans* encoded CRISPR-I system required for RliB-CRISPR DNA interference activity, as shown in *L. monocytogenes* Finland strain. However, the conservation of the RliB-CRISPRs in *Listeria* is independent on the presence of CRISPR-I, strongly suggesting that it is a functional element with an important function even in the absence of Cas Type-I, as sequences lacking selection pressure for their maintenance are quickly lost in bacterial genomes [40]. Interestingly, RliB-CRISPRs in average possess a smaller number of repeats and the variability of their spacers is lower compared to the spacer content of the CRISPR-I and CRISPR-II. Have they evolved a new function? It is to be kept in mind the remarkable finding that all RliB-CRISPRs accumulate as a 280 nt fragment, which might be the functional form. In support for a functional role of RliB, our recent RNA-seq analysis has shown that RliB-CRISPR is not only conserved but also expressed in the more distant *L. innocua* species that also lacks CRISPR-I [41].

Although RliB-CRISPRs share many similarities with *cas*-associated CRISPR-I system, the identified RliB-CRISPR protospacers are more often in the antisense orientation with respect to the corresponding spacer and in addition they are mostly located in the late phage genes encoding DNA packaging and envelope proteins. It is tempting to speculate that “the” RliB-CRISPRs *cas*-independent activity might be RNA interference. In this scenario RliB-CRISPR would not destroy the bacteriophage DNA but would rather control the bacteriophage late gene expression i.e., it would prevent the formation of viral particles and lysis of the bacterial cell. RliB-CRISPR interference could be also based on transcription-dependent DNA targeting, as recently described in *Sulfolobus islandicus* REY15A [42]. Alternatively, RliB-CRISPR might have evolved a broader function relevant for *Listeria* physiology that is not related to the immunity. Such examples have been described in *Pseudomonas aeruginosa*, where a CRISPR appear to be involved in lysogeny dependent biofilm formation [43], in myxobacteria where CRISPR has been implicated in swarming motility [44] and more recently in *Francisella novicida* where a tracrRNA was shown to regulate an endogenous transcript encoding a lipoprotein important for the bacterial infection [45].

The interaction between bacteriophages and bacteria is mostly seen as a parasitic interaction where the virus exploits the host resources for its own benefit. However, there are some viruses that have a beneficial effect on their host [46]. In case of pathogenic bacteria, bacteriophages often carry virulence factors required for successful infection [47]. More recently, a study by Rabinovich et al. (2012) showed that during *Listeria* intracellular infection, a temperate prophage is excised, which reconstitutes a function of the gene where the bacteriophage was integrated, and promotes bacterial escape from macrophage phagosomes. Remarkably, the excision event does not lead to propagation and release of the progeny virions neither to the subsequent lysis of the bacterial cell. Hence, the virion production is actively aborted [48]. This example highlights an important crosstalk between the phage and the pathogenic bacteria during the infection of the mammalian cell, and more importantly, it emphasizes the conditional advantage for a bacterium to maintain a bacteriophage and control its virulence. Our previous studies have shown that RliB expression is upregulated in the bacteria grown in human blood and in the intestine of gnotobiotic mice and is important for *Listeria* virulence [4]. Whether RliB-CRISPR expression and prophage excision followed by aborted virion production are linked processes, remains to be examined. Our study thus paves the way for new regulatory studies on the interactions between bacteriophages and bacteria during saprophytic life or during infection.

## Materials and Methods

### Strains and plasmids

Strains used in this study are *L. monocytogenes* EGD-e (BUG1600) and its isogenic mutants Δ*rliB*(BUG2621) and Δ*pnpA*(CMA751), *L. monocytogenes* EGD (BUG600) and its isogenic mutant, Δ*pnpA-EGD* (BUG3415) and Δ*rliB-EGD* (BUG3243), *L. monocytogenes* Finland 1998 (BUG3297, CLIP2012/00396, FE49845/IHD42536) and its isogenic mutants Δ*pnpA-Fin* (BUG3465) and Δ*rliB-Fin* (BUG3466). Mutants were obtained by deletion of the corresponding ORF or non-coding RNA by PCR-ligation and amplicon cloning in the suicide vector pMAD as previously described [49]. Overexpression of RliB was obtained by cloning the *rliB* gene into the pAD vector carrying Phyper constitute promoter [50], resulting in the strain Phyper-RliB (BUG2987). PNPase complementation was obtained by cloning the PnpA ORF into the pPl2 vector [51] resulting in the strain Δ*pnpA*+pnpA (CMA752).

### Bacterial growth

Bacteria were grown overnight in Brain heart infusion (BHI) medium (Difco) at 37°C with shaking at 200 rpm. Cultures were subsequently diluted 1/500 into 100 ml BHI and grown at 37°C until mid-exponential phase (OD_600_ = 1.0). When required, erythromycin and chloramphenicol were used at 5 µg/ml and 20 µg/ml, respectively as final concentration. For RNA extraction, bacteria were pelleted, by centrifugation at 10,000 X G for five minutes, flash frozen in liquid nitrogen and stored at −80°C.

### RNA isolation

Bacterial pellets were resuspended in 400 µl solution A (½ volume Glucose 20%+½ volume Tris 25 mM pH 7.6+EDTA 10 mM) to which an additional 60 µl of 0.5M EDTA was added. Bacteria were lysed in FastPrep homogenizer (Bio101) and RNA was subsequently extracted using TRI reagent (Invitrogen) as described previously [4]. RNA integrity was verified using the Experion Automated Electrophoresis system (Biorad).

### Northern blot analysis

10–20 µg of total RNA was mixed with two volumes of Formaldehyde Loading Buffer (Ambion) followed by denaturation at 65°C for 15 min. Samples were separated by electrophoresis on 5% TBE-Urea polyacrylamide gels (Criterion-Biorad) at 100 V for 2 hours in 1× TBE running buffer at RT, followed by an overnight transfer at 4°C/100 mA to Nytran membranes (Sigma). Membranes were UV-crosslinked and probed with RNA probes or DNA oligo probes. Briefly, RNA probes were synthesized and α^32^P-UTP labelled using the Maxiscript T7RNA polymerase kit (Ambion) with PCR generated templates according to the manufacturer's instructions. Oligonucleotide DNA probes were 5′ labelled with γ^32^P-ATP using the T4 Polynucleotide Kinase according to the manufacturer's protocol (New England Biolabs). Membranes were prehybridized for 60 min in Ultrahyb buffer (Ambion) and hybridizations were performed overnight at 64°C for RNA probes and at 37°C for oligonucleotide probes. Following hybridization, membranes were washed twice for 5 min with 2× SSC, 0.1% SDS at room temperature. When hybridized with RNA probes, membranes were additionally washed twice for 15 min in 0.1× SSC, 0.1% SDS at 60°C. The size marker was a 50-bp ladder (Invitrogen), which was 5′ end labelled with γ^32^P-ATP.

### Affinity chromatography using MS2-RliB or biotinylated RliB

We first have optimized an affinity purification assay using 3′-biotinylated RliB and streptavidin sepharose modified as described in Jestin et al. [52]. As a negative control, we used the regulatory RNAIII from *S. aureus*. Total cell extract prepared from 500 ml of culture of *L. monocytogenes* Δ*rliB* mutant strain was first incubated with streptavidin sepharose beads to remove proteins unspecifically bound to the beads. The beads were first incubated with the 3′ biotinylated RNA and the pre-cleaned crude extract was passed through the column and washed with the binding buffer containing 50 mM Hepes-NaOH pH 7,5, 5 mM MgCl_2_, 1 mM DTT, and 150 mM KCl. The elution of the proteins was done with the same buffer containing 6 M urea, 2 M thiourea and 30 mM d-biotin. The fractions were then analyzed by 4–15% gradient SDS-PAGE, and the proteins were identified by mass spectrometry.

A second approach was used to purify proteins associated with RliB carrying at its 5′ end two hairpin motifs recognized by the coat protein of the MS2 bacteriophage. As a control we used the untagged RliB. Both RNAs were transcribed *in vitro* using homemade T7 RNA polymerase. The experimental conditions were as previously described [53]. The MS2 coat protein fused to Maltose binding protein was expressed in *E. coli* and purified on an amylose column followed by a monoQ column. The MS2-MBP coat protein was first immobilized on the amylose resin, and the tagged-RNA was loaded on the column, which was washed with 2 ml of the Binding Buffer. Subsequently, the pre-cleared bacterial lysate was loaded onto the column, followed by three washes with 2 ml Binding Buffer, and the proteins were eluted with the binding buffer containing 10 mM maltose. The fractions were loaded on a SDS-PAGE and the proteins were identified by mass spectrometry.

### RNA structure probing

Enzymatic hydrolysis was performed with 1 pmol of RliB in 10 µl of a buffer containing 50 mM NaOH-Hepes pH 7.5, 10 mM MgCl_2_, 150 mM KCl, in the presence of 1 µg carrier tRNA at 20°C for 5 min: RNase T2 (0.01 units), RNase V1 (0.5 units). Chemical modifications were performed on 2 pmol of RliB at 20°C in 20 µl of the same buffer containing 2 µg of carrier tRNA. Methylation of C(N3) and A(N1) positions was done with 1 µl DMS (diluted 1/8 and 1/16 in ethanol) for 2 min at 20°C. Modification of U(N3) and G(N1) was performed with 2,5 µl and 5 µl of CMCT (40 mg/ml) for 20 min at 20°C. The cleavage or modification sites of unlabeled RNAs were detected by primer extension. Details for hybridization conditions, primer extension, and analysis of the data have been previously described [54].

### PNPase cleavage assays

PNPase cleavage assays were done using a 5′ end-labelled RliB or RNA37. Reaction was performed in 10 µl of TMK buffer containing 20 mM Tris-HCl pH 7.5, 10 mM magnesium-acetate, 100 mM KCl, 1 mM DTT at 37°C for 15 min in the presence of PNPase 200 nM in the presence of 1 µg of carrier tRNA. Competition experiments were carried out in the presence of 200 nM, 400 nM of cold RliB and its derivatives (RliB-3′ domain or RliB-5′ domain). Reactions were stopped by phenol extraction followed by RNA precipitation. The assays were loaded on a denaturing 12% polyacrylamide-urea gel electrophoresis. The PNPase cleavage sites were assigned by running in parallel RNase T1 ladder and an alkaline ladder on a denatured end-labelled RNA [54].

### Gel retardation assays

To perform gel retardation assays, 5′ end-labelled transcript (20000 cpm, <1 nM) was incubated in the presence of increasing concentrations of PNPase (100 to 800 nM) in TMK buffer containing 20 mM Tris-HCl pH 7.5, 10 mM magnesium-acetate, 100 mM KCl at 37°C for 15 min. At the end of the binding reaction 6X loading dye (30% glycerol, 0.25% bromophenol blue and 0.25% xylene cyanol) was added to the samples and they were analyzed on a 6% polyacrylamide gel under non-denaturing conditions.

### CRISPR activity assay

#### Plasmid design

The DNA fragment of the protospacer plasmid (P) was reconstituted using two complementary, 5′-phosphorylated oligonucleotides with the flanking BamH-I restriction sites P-A (GATCCGGTCGTCCTGCGATTTTTGTCAAAGGGACAGCGATGGGTTACAAGGAATACG) and P-B-(GATCCGTATTCCTTGTAACCCATCGCTGTCCCTTTGACAAAAATCGCAGGACGACCG) whereas the DNA fragment of the control plasmid was reconstituted using the oligonucleotides C-A (GATCCGGTCGTCCTTAAGTTTTATAACCATGGAAGTTGGAGCGAGCCCGGGAATACG) and C-B (GATCCGTATTCCCGGGCTCGCTCCAACTTCCATGGTTATAAAACTTAAGGACGACCG). 45 µl (c = 100 µM) of each oligonucleotide was added to 10 µl NEB buffer 3. The mixture was heated to 95°C and then slowly cooled down to room temperature. The reconstituted DNA fragments were cloned to Ppl2 integrative vector [51] using the BamH-I restriction site. Obtained clones were isolated and sequenced in order to verify the sequence integrity and the orientation of the cloned fragment. Plasmids with only one orientation of the fragments P and C were used for all the performed experiments.

#### Conjugation

Plasmids P and C were heat shock transformed to a donnor *E. coli S17* to create *E.coli-P* and *E.coli-C* strains. Overnight cultures of *E. coli* and *L. monocytogenes* strains were diluted 1/20 in 5 ml LB media supplemented with 35 µg/ml chloramphenicol and 5 ml BHI media, respectively. The cultures were grown until mid exponential phase (OD = 1.0). 1 ml of each *E. coli* culture was washed 3X with 1 ml BHI media to remove the remaining antibiotic. The equal amount of *E. coli-P* and *E. coli-C* cultures were mixed. Subsequently, 200 µl of *E. coli-P/C* mixture was added to 200 µl *L. monocytogenes* culture and placed on the milipore filter (0.45 mm) on top of the agar plate and incubated overnight at 37°C. The next day, the cultures were removed from the filter, resuspended in 1 ml of BHI and plated on BHI plates supplemented with 7 µg/ml chloramphenicol, 50 µg/ml nalidixic acid and 100 µg/ml colistin. The plates were incubated 72 h at 37°C.

#### Quantitative PCR (Q-PCR)

Colonies were picked on a new BHI plate supplemented with 7 µg/ml chloramphenicol and incubated overnight at 37°C. The next day, each colony was resuspended in 50 µl water and heated to 95°C during 20 min. After centrifugation, supernatant was used as the template for the Q-PCR. Oligonucleotides were used to specifically amplify the fragments in the P plasmid (P-fw CAGGGCAGGGTCGTTAAATAG and P-rev AGCGATGGGTTACAAGGAATAC) and C plasmid (C-fw CGACTCACTATAGGGCGAATTG and C-rev CGCTCCAACTTCCATGGTTAT). As the control, we used oligonucleotides corresponding to the chloramphenicol gene present in both plasmids (Cm-fw CACTCATCGCAGTACTGTTGTA and Cm-rev CAAACGGCATGATGAACCTG). The reaction was based on Sso Fast Eva Green Supermix (Biorad) using the CFX384 Real-Time System (Biorad). The Ct values corresponding to P or C specific amplification were compared to deduce the plasmid identity of each individual colony. To evaluate the *E.coli-P/C* conjugation mix and the levels of the plasmids P and C at the beginning of the experiment, the plasmids from the *E.coli-P/C* mix were extracted using the MiniPrep Kit (QIAGEN) and their relative quantity was compared using the Q-PCR with the aforementioned oligonucleotides. To calculate the ratio (R = n(P)/n(C)), at least 32 colonies of each strain were screened. Each experiment was repeated multiple times on independent conjugation reactions. Results were statistically analyzed using t-tests.

### Bioinformatic analysis

We analyzed 45 *Listeria* genomes taken from GenBank, available at the time of analysis. These include 28 complete genomes and 17 draft genomes with less than 800 contigs (Table S1). We also added the genome of *L. monocytogenes* EGD strain (unpublished data). We used GenBank annotations, excluded genes with stops in phase and with lengths not multiple of three. We re-annotated the prophages in the genomes using a methodology described previously [55].

#### Cas gene identification

The Hidden Markov models (HMMs) for the Cas protein families described previously [6] were obtained from the TIGRFAM database, version 6.0 (http://www.tigr.org/TIGRFAMs/). To identify *cas* genes, all coding sequences within each complete and draft genomes were searched with the 57 Cas HMMs profiles using hmmpfam [56] (e-value <0.001) (Table S1). To identify *cas* pseudogenes, all Cas proteins previously detected were searched in all the genomic sequences using tblastn (e-value <0.001).

#### Identification of the core genome

A preliminary set of orthologs was defined by identifying unique pairwise reciprocal best hits, with at least 60% similarity in amino acid sequence and less than 20% of difference in protein length. The list was then refined using information on the distribution of similarity of these putative orthologs and data on gene order conservation (as in [57]). The analysis of orthology was made for every pair of *L. monocytogenes* and *L. innocua* genomes. The core genome was defined as the intersection of pairwise lists of positional orthologs and was used to build the phylogenetic tree (see below). We used *L. innocua* as out-group to root the tree of *L. monocytogenes*.

#### Phylogenetic analysis

The reference phylogenetic tree for the core genome of the 43 complete/draft genomes was reconstructed from the concatenated alignments of 513 proteins of the core genome obtained with muscle v3.6 [58] then back-translated to DNA, as is standard usage. We used Tree-puzzle 5.2 [59] to compute the distance matrix between all genomes using maximum likelihood under the HKY+G(8)+I model. The tree of the core genome was built from the distance matrix using BioNJ [60]. We made 100 bootstrap experiments on the concatenated sequences to assess the robustness of the topology. The topology of this tree is congruent with previous

#### CRISPR array identification

CRISPR arrays were identified using CRT (CRISPR Recognition Tool) with default parameter values [61], in all the *Listeria* complete/draft genomes publicly available at the time of analysis. In the complete genomes, loci bordered by the same core genes were identified as RliB-CRISPR (bounded by the core genes: *lmo0509*-*lmo0510*), CRISPR-I (*lmo0517*-*lmo0518*) and CRISPR-II (*lmo2951*-*lmo2596*) (Figure 4). For each array, the repeats were extracted and were aligned using Muscle [58]. Then we used Cons (http://bioweb.pasteur.fr/docs/EMBOSS/cons.html) to obtain consensus sequences from these multiple sequence alignments of the three arrays. In all cases, the consensus sequence corresponds to the most frequent sequence within a particular array. We used the sequence of the repeats as patterns to identify additional, smaller and/or degenerate repeat clusters in all available complete and draft genomes. This analysis was done with Fuzznuc (http://bioweb2.pasteur.fr/docs/EMBOSS/fuzznuc.html).

#### Protospacer identification

Blastn was used for similarity searches between CRISPR spacer sequences and all the complete prokaryote genomes (3703 replicons), complete plasmid genomes (2930), and virus genomes (1153) available in GenBank. Matches showing an E value lower than 10^−5^ and less than 10% difference in sequence length between query and hit were retained. Matches to sequences found within CRISPR loci were ignored.

## Supporting Information

Figure S1 Structure probing of RliB using chemicals and enzymes. The secondary structures of the full-length RliB (RliB) and RliB carrying the MS2 tag at its 5′ end (RliB-MS2) were probed using reverse transcription after chemical modification and enzymatic hydrolysis. Lane 1: incubation controls; lane 2: DMS modification to probe position N1 of adenines and N3 of cytosines; lanes 3–4: CMCT modifications to probe position N3 of uridines and N1 of guanines; lanes 5–6: RNase V1 hydrolysis to map double-stranded regions of RNA; lanes 7–8: RNase T2 hydrolysis to map the unpaired regions of RNA (with a preference for adenines). Lanes A, U, G, C: dideoxy-sequencing reactions performed on RliB-MS2 mRNA.
(EPS)

Figure S2 Putative secondary structure of representative RliB-CRISPRs. The secondary structure of the full length RliB-CRISPR containing different number of repeats (red) and spacers (black): A) RliB-CRISPR containing 10 spacers conserved in *L. monocytogenes* EGD, 10403S, SLCC5850 strains, B) RliB-CRISPR containing 6 spacers conserved *in L. monocytogenes* F6900, J2818, F6854, J0161, C) RliB-CRISPR containing 3 spacers conserved in all analyzed *L. monocytogenes* strains belonging to lineage I.
(EPS)

Figure S3 Comparison of the putative leader sequences of RliB-CRISPR and CRISPR-I. A) Multiple alignments of putative leader sequences preceding identified RliB-CRISPRs and CRISPR-I among analyzed *L. monocytogenes* strains. The transcription start site of the RliB-CRISPR in *L. monocytogenes* EGDe strain is indicated and the beginning of the repeat sequence. B) Logo and the consensus sequence generated from the multiple alignment. Highlighted is conserved putative RpoD dependent promoter.
(EPS)

Figure S4 *Listeria* prophages. A) 29 complete *Listeria* genomes are represented. In the line with each genome is indicated presence of a prophage. The prophages are positioned on the vertical grid according to their integration site in the *Listeria* genome (tRNA-Lys, tRNA-Ser, tRNA-Arg, 678, EF-Ts, 1041, comK, tRNA-Arg, tRNA-Thr, 1681). Prophages were identified according to their similarity with the bacteriophage sequences available in the Genbank. A prophage is assigned with a name if it shares at least 40% similarity with a known phage and is highlighted as a dot with the corresponding colour (red-B054, blue-B025, green-A118/A500, yellow-A006, purple-PSA). B) Sequence alignments between the RliB/CRISPR spacer (S7) and three CRISPR-II spacers (S21, S22, S23) in the *L. monocytogenes* EGD strain that match B025 prophage integrated in the EGD chromosome. The numbers on the left indicate the exact position of the protospacer within the EGD genome whereas match and identity percentage are highlighted above.
(EPS)

Figure S5 Protospacer-adjacent motifs (PAMs) of RliB-CRISPR, CRISPR-I and CRISPR-II. Protospacer-adjacent motifs (PAMs) were generated by multiple alignments of the regions flanking the identified protospacers. The analysis was done separately for each CRISPR system (RliB-CRISPR, CRISPR-I and CRISPR-II) distinguishing those derived from prophages identified among *Listeria* strains and bacteriophages present in the GenBank. Above each PAM motif is indicated a number of protospacers used for the Logo production.
(EPS)

Figure S6 Position of the protospacers along the bacteriophage genomes. The positions of the protospacers along the *Listeria* bacteriophage genomes are shown. The colours correspond to different homologous gene families. Two proteins were considered as homologous if they have at least 60% of similarity in amino acid sequence and less than 20% of difference in protein length. Arrows indicate position of protospacers identified by the similarity to the spacers composing RliB-CRISPR (green), CRISPR-I (blue) and CRISPR-II (red). A) Genomes of 6 temperate bacteriophages (B054, B025, PSA, A500, A118 and A006) are shown. Bacteriophage genes encoding proteins involved in DNA packaging and structural proteins are highlighted. B) Genomes of 4 virulent bacteriophages (P70, P35, P100 and A115) are shown.
(EPS)

Table S1 Presence of the CRISPR/Cas system in available complete and draft *Listeria* genomes (provided as an Excel table).
(XLSX)

Table S2 List of spacers analysed in this study (provided as an Excel table).
(XLSX)

Table S3 Summary of the spacer composition of CRISPR arrays in *Listeria* (provided as an Excel table).
(XLSX)

Table S4 Description of the unique spacers of the CRISPR-I targeting phage and prophage regions (provided as an Excel table).
(XLSX)

Table S5 Description of the unique spacers of the CRISPR-II targeting phage and prophage regions (provided as an Excel table).
(XLSX)

Table S6 Description of the unique spacers of CRISPR RliB targeting phage and prophages regions (provided as an Excel table).
(XLSX)

Table S7 Self-targeting spacers (provided as an Excel table).
(XLSX)
